# Association of Pro-Inflammatory Cytokines and Iron Regulatory Protein 2 (IRP2) with *Leishmania* Burden in Canine Visceral Leishmaniasis 

**DOI:** 10.1371/journal.pone.0073873

**Published:** 2013-10-11

**Authors:** Paulo Ricardo Porfírio do Nascimento, Daniella Regina Arantes Martins, Glória Regina Góis Monteiro, Paula Vivianne Queiroz, Francisco Paulo Freire-Neto, José Wilton Queiroz, Ádila Lorena Morais Lima, Selma Maria Bezerra Jeronimo

**Affiliations:** 1 Department of Biochemistry, Bioscience Center, Federal University of Rio Grande do Norte, Natal, Rio Grande do Norte, Brazil; 2 Institute of Tropical Medicine of Rio Grande do Norte, Federal University of Rio Grande do Norte, Natal, Rio Grande do Norte, Brazil; 3 Department of Biology and Genetics, Bioscience Center, Federal University of Rio Grande do Norte, Natal, Rio Grande do Norte, Brazil; 4 Health Graduate Program, Health Science Center, Federal University of Rio Grande do Norte, Natal, Rio Grande do Norte, Brazil; 5 College of Medicine, State University of Rio Grande do Norte, Mossoró, Rio Grande do Norte, Brazil; 6 Institute of Science and Technology of Tropical Diseases (INCT-DT), Salvador, Bahia, Brazil; Washington State University, United States of America

## Abstract

*Leishmania infantum* infection in humans and dogs can evolve with a wide range of clinical presentations, varying from asymptomatic infections to visceral leishmaniasis. We hypothesized that the immune response elicited by *L. infantum* infection could modulate whether the host will remain asymptomatic or progress to disease. A total of 44 dogs naturally infected with *L. infantum* were studied. *Leishmania* burden was estimated in the blood and spleen by qPCR. The expression of *IFN-γ*, *TNF-α*, *IL-10* and Iron Regulatory Protein 2 (IRP2) were determined in the spleen by quantitative PCR. Sera cytokines were evaluated by ELISA. Dogs were grouped in quartiles according parasite burden. Increased expression of *IFN-γ* and *TNF-α* was associated with reduced *Leishmania* burden, whereas increased *IL-10* and *IRP2* expressions were associated with higher *Leishmania* load. Increased plasma albumin and *IFN-γ* expression explained 22.8% of the decrease in parasite burden in the spleen. These data confirm that lower IFN-γ response and higher *IL-10* correlated with increased parasite load and severity of the visceral leishmaniasis in dogs. The balance between the branches of immune response and the intracellular iron availability could determine, in part, the course of *Leishmania* infection.

## Introduction

*Leishmania infantum* infection can result in a wide range of clinical outcomes, varying from self-resolving infection to visceral leishmaniasis, which can be fatal, even with treatment [1]. Visceral leishmaniasis (VL) used to be reported in sporadic manner in rural areas of Northeast region of Brazil and children had a greater risk of developing symptomatic disease [1,2]. However, in the last 20 years VL became a disease of perimetropolitan areas of major cities in Brazil [3-6] and has spread to other countries in Latin America [7]. These changes have resulted in a large population at risk of *L. infantum* infection. Anemia and opportunistic co-infections are usually the main features found in in visceral leishmaniasis [8-10]. More recently, co-infection with HIV has become an important risk for human VL and about 5% of VL cases in Brazil are diagnosed in people with AIDS [11]. Mal nutrition is also associated with risk of visceral leishmaniasis [1,2,9]. Children with symptomatic VL in Bangladesh had lower serum levels of retinol and zinc compared to uninfected and asymptomatically infected people [12].

Similar to humans, dogs infected with *L. infantum* can also evolve with a spectrum of clinical manifestations [13]. Canine visceral leishmaniasis (CVL) is characterized by lymphadenopathy, onychogryphosis, cutaneous lesions, cachexia, weight loss and pancytopenia [14]. Asymptomatic dogs often progress to disease [15]. Inversion in albumin/globulin ratio, increased anti-*Leishmania* antibodies titers and increased IL-10 production are associated with disease [13]. Dogs in Brazil are considered the main reservoir of *Leishmania*, and major efforts for disease control have been focused on the euthanasia of infected animals. However, those actions have not been effective [11], mostly because there is usually a lag between infection ascertainment and the placement of the control measures. 

Iron is an important micronutrient acquired from the diet and is a central component of haem groups, in mitochondrial proteins and co-factors for many enzymes involved in anti-oxidant responses [16,17]. However, iron is extremely toxic to the cell in its reduced form (Fe^2+^), through increase in hydroxyl radicals, via Fenton’s reaction, which may damage organelles, DNA or cell membranes [18]. In order to regulate intracellular iron contents, mammalian cells developed an elegant mechanism capable to finely regulating intracellular iron levels, through expression of Iron Regulatory Protein 2 (IRP2), an intracellular iron sensor. In iron depleted condition IRP2 changes its conformation and binds to transferrin receptor (Tfr) mRNA, allowing translation, and increase cellular iron intake [19,20]. In this current study, we found that dogs with lower parasite burden expressed more *IFN-γ* and *TNF-α*; conversely, animals with higher *Leishmania* burden had lower *IFN-γ* and *TNF-α* expressions and higher expression of *IRP2* and *IL-10* in the spleen. These findings reinforce that *IFN-γ* and *TNF-α* expression are involved in the control of parasite and they are down modulated by the increased *IL-10* expression.

## Materials and Methods

### Studied dogs

Blood and spleen samples from 44 *L. infantum*-infected dogs (21 males and 23 females) age ranging from 3 to 8 years old, were obtained from the municipal Center for Zoonotic Control from Natal, Rio Grande do Norte (RN), Brazil, where the animals were euthanized. Dogs have been presumed to be the main *Leishmania* reservoir in Brazil [11,13] and as part of leishmaniasis control program infected animals are euthanized. *L. infantum* infection was determined by indirect immunofluorescence test (IFAT), assay performed at the Center for Zoonotic Control, and a title>1:40 was considered positive. Anti-Leishmania antibodies were determined by ELISA using soluble Leishmania antigen (SLA) obtained from an isolate of a human patient with VL from Natal, Rio Grande do Norte, Brazil (IOC 563) or rK-39 *Leishmania* antigen, as previously described [21], but using as secondary antibody anti-dog IgG (Bethyl laboratories, Montgomery, TX, USA). The cut-off values was of optical density of 0.096 and 0.112 for anti-SLA and anti-rK-39, respectively, were determined based on the mean results of samples from 6 dogs from a non-endemic VL area + 3 standard deviation of the mean.

### Ethical Considerations

The study protocol was assessed and approved by the Animal Research Ethics Committee of Federal University of Rio Grande do Norte (CEUA-UFRN), under the number 012/2010. Dogs were euthanized by the Center for Zoonotic Control from Natal. All procedures involving the animals were conducted according to the guidelines of the Brazilian College for the usage of animals in Experiments (COBEA). 

### IgG1 and IgG2 subclasses determination

Anti-SLA IgG1 and IgG2 were determined by ELISA as previously described by Braz et al. [21] with modifications, but with canine anti-IgG1 and anti-IgG2 peroxidase conjugated used as secondary antibodies (Bethyl laboratories, Montgomery, TX, USA). 

### Leishmania culture

Spleen homogenate was incubated in modified minimum essential medium (HOMEM) with 10% heat inactivated fetal calf serum and hemin (8 µM). Culture was examined at light microscope every 72 hours. This procedure was carried out until promastigotes were visualized or up to 30^th^ day after initial culture. *Leishmania* isolates were typed at reference World Health Organization Laboratory (Elisa Cupolillo, PhD, Fiocruz Foundation, Rio de Janeiro, RJ, Brazil). All isolates were typed as *L. infantum*. The typing was also confirmed by using specific primers for *Leishmania*, as described by Weirather et al [22].

### Serum albumin concentration

Serum samples were obtained from each dog and diluted 1:100 in phosphate buffered saline (PBS). Albumin was determined by spectrometry at 630 nm and the concentration was calculated according to manufacturer instructions (Labtest, Lagoa Santa, MG, Brazil). 

#### RNA extraction and cDNA synthesis

Total RNA was extracted from 100 mg of spleen using Trizol (Invitrogen, Grand Island, NY, USA). RNA pellet was suspended in nuclease-free water (Ambion, Grand Island, NY, USA). Samples were treated with DNase for 30 minutes at 37°C (Invitrogen, Grand Island, NY, USA) and RNA quality was verified by agarose gel electrophoresis. Reverse transcription was performed with High Capacity cDNA Reverse Transcription Kit (Applied Biosystems, Foster City, CA, USA), and was carried out at 37°C for 120 minutes followed by an incubation at 75°C for 5 minutes. 

### DNA extraction

*L. infantum* in the spleen and in the peripheral blood were estimated by quantitative PCR [22]. DNA was extracted from blood (5ml) and from spleen (100 mg) by adding 20 mL of red blood cells lyses buffer [(NH_4_)_2_CO_3_ 100 mM; NH_4_Cl 10 mM] to 5 mL of blood or to spleen homogenate. The homogenates were incubated for 10 minutes at room temperature and centrifuged at 3.200 g, with this procedure repeated twice. The white cell pellet was then lysed with 5 mL of lysis buffer (Tris 100 mM; EDTA 25 mM; SDS 10 mM), digested with proteinase K (20 µl at 25 mg/mL) (Invitrogen, Grand Island, NY, USA), and incubated overnight at 55°C. A total of 3 mL of ammonium acetate 5 M (C_2_H_7_NO_2_) were added, incubated at -20°C for 5 minutes, followed by centrifugation (3.200 g for 10 minutes) and then the supernatant was transferred to a new tube containing 8 mL of isopropyl alcohol, samples were vigorously mixed until the DNA was visualized. DNA was transferred to a new tube and washed with 75% ethanol. DNA was suspended in water. DNA concentration was measured at 260 nm.

### Cytokine and IRP2 expression in the spleen

Level of *IFN-γ, TNF-α, IL-10* and *IRP2* expressions were determined in the spleen by quantitative PCR (qPCR), with *β-Actin* (ACTB) taken as endogenous control. The set of primers and probes used are shown in Table S1. q-PCR was carried out in a final volume of 10µL in 7500 Real Time PCR System (Applied Biosytems, Foster City, CA, , USA). The reaction was carried out with an initial denaturation step of 10 minutes at 95°C, followed by 40 cycles of denaturation, for 15 seconds, at 95°C, and annealing/extension for 1 minute, at 65°C. Relative gene expression was analyzed by ΔΔCt method [23] taking the results obtained from dogs belonging to quartile 1 were used as the calibrator group, since in our setting it was hard to find uninfected dogs. 

### IFN-γ, TNF-α and IL-10 level in the sera

IFN-γ, TNF-α and IL-10 levels in the sera were determined by Sandwich-ELISA (R&D Systems, Minneapolis, MN, USA). 50 µL of serum, positive controls and standards were loaded on a 96-wells plate pre-coated with anti-canine-IFN-γ, TNF-α or IL-10. Plates were incubated for 2 hours, at room temperature, followed by washing and then the addition of the secondary antibody (2 hours at room temperature). The excess of unbound antibodies were removed by washing, followed by addition of the substrate solution (100 µL per well), incubation (30 minutes at room temperature) and then the Stop Solution was added to each well (100 µL). The optical density was determined using a microplate reader (Bio-Tek Instruments, Winooski, VT, USA) at 450 nm with correction at wavelength 540 nm. A standard curve was obtained and the serum concentration of the cytokines was inferred. 

### Quantification of *Leishmania* by quantitative PCR

To estimate the parasite load in the spleen and in the blood, kinetoplast *L. infantum* DNA (k-DNA) was amplified using a set of primers and probe as previously reported [22]. Primes included forward 5’CTT TTC TGG TCC GGG TAG G 3’; Reverse 5’ CCA CCC GGC CCT ATT TTA CAC CAA 3’; and Probe 5’/56 FAM/ TTT TCG CAG AAC GCC CCT ACC CGC 3’TAMRA (Integrated DNA Technologies, IDT Inc. Coralville, IA, USA). A standard curve was generated from DNA extracted from a known number of cultured *L. infantum* promastigotes from a local isolate, obtained from a patient with VL, ranging from 10^6^ to 10^-1^ parasites per well. PCR was carried out in a final volume of 10 µL, containing 200 nM of reverse and forward primers and FAM-probe. The qPCR standard curves had a mean R^2^ of 0.992, a slope of -3.9 and an efficient % of 75.35. A total of 80 ng of peripheral blood or spleen DNA were used as template and 5 µL of TaqMan® Universal PCR Master Mix. The reaction was carried out in a 7500 real time PCR system (Applied Biosystems, Foster City, CA, USA) were used. 

### Statistical analysis

Animals were grouped in quartiles according to the range of spleen k-DNA Ct and 4 groups were considered for analysis purpose: Q1, with spleen parasite load estimated in 27,024±7,826 parasites/spleen mg (n=11); Q2, presenting a spleen parasite load of estimated in 84,399±36,524 parasites/spleen mg (n=12); Q3, whose has the spleen parasitism estimated in 205,074±54,172 parasites/spleen mg (n=10); Q4, which has a spleen parasite load estimated in 485,192±125,661 parasites/spleen mg (n=11), the equivalent of parasites per Ct is shown Table S2. Data were first analyzed for normality using Kolmogorov-Smirnov test. Paired data were compared by T test or Mann-Whitney. Multiple comparisons were performed using ANOVA or Kruskal-Wallis test, followed by Dunns post-test. A linear regression model, with backward selection method [24], was built to assess the relation of cytokines (IFN-γ, TNF-α, IL-10), *IRP*2 expression, anti-*Leishmania* antibodies and serum albumin on spleen parasitism.A Pearson Matrix correlation was obtained to all variables. A p-value <0.05 was considered significant. 

## Results

### Clinical characteristics of the studied dogs

**Figure 1:**
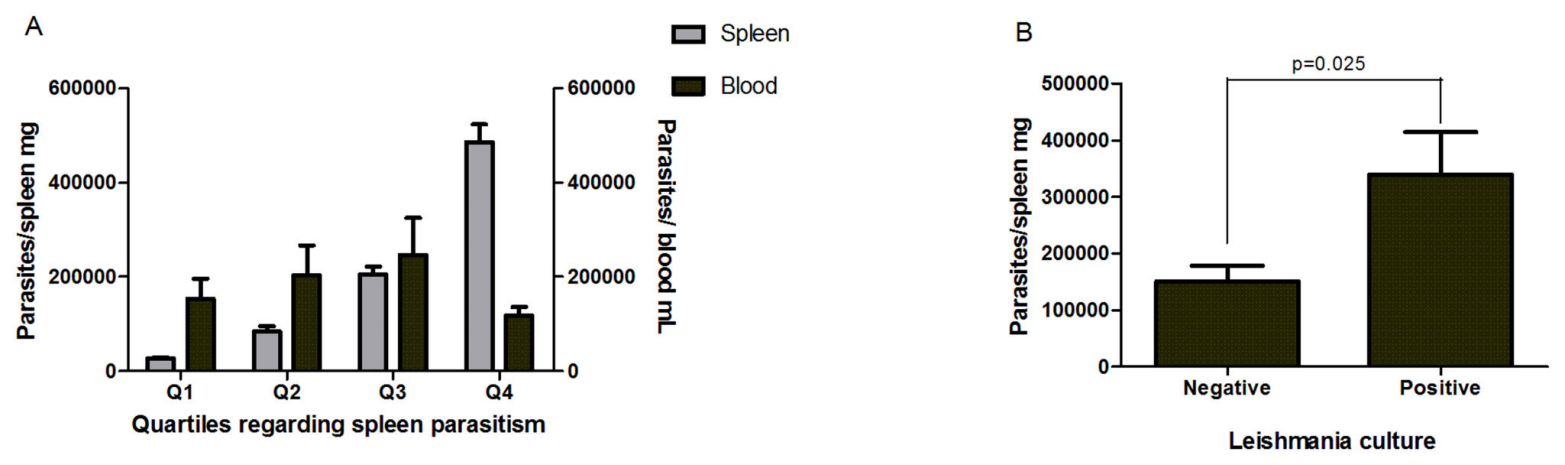
*Leishmania* load in the blood and the spleen of dogs naturally infected with *Leishmania infantum*. A. Dogs were grouped in quartiles according to spleen parasite burden and levels were compared to *Leishmania* level in the blood. Analysis was performed Mann-Whitney test. The Kolmogorov-Smirnov test was used to test the normality of the data. B. Dogs were grouped in accordance to *Leishmania* culture, positive or negative.

Of the 44 dogs, 21 were males and 23 were females (p>0.05). All animals had anti-*Leishmania* antibodies (0.9 ± 0.64 for anti-SLA, and 0.9 ± 0.6 for anti-k39). Of the 44 animals, 33 (75%) were culture positive for *Leishmania*, but *Leishmania* DNA was amplified from the spleen and blood of all animals, by using specific primers for *L. infantum* [22]. Dogs whose spleen samples were culture negative for *Leishmania* had lower spleen parasitism than those cultured positive (p=0.025; Figure 1A). The range of clinical manifestations varied from apparent healthy dogs either with or no skin lesions to severe malnourished.

**Figure 2:**
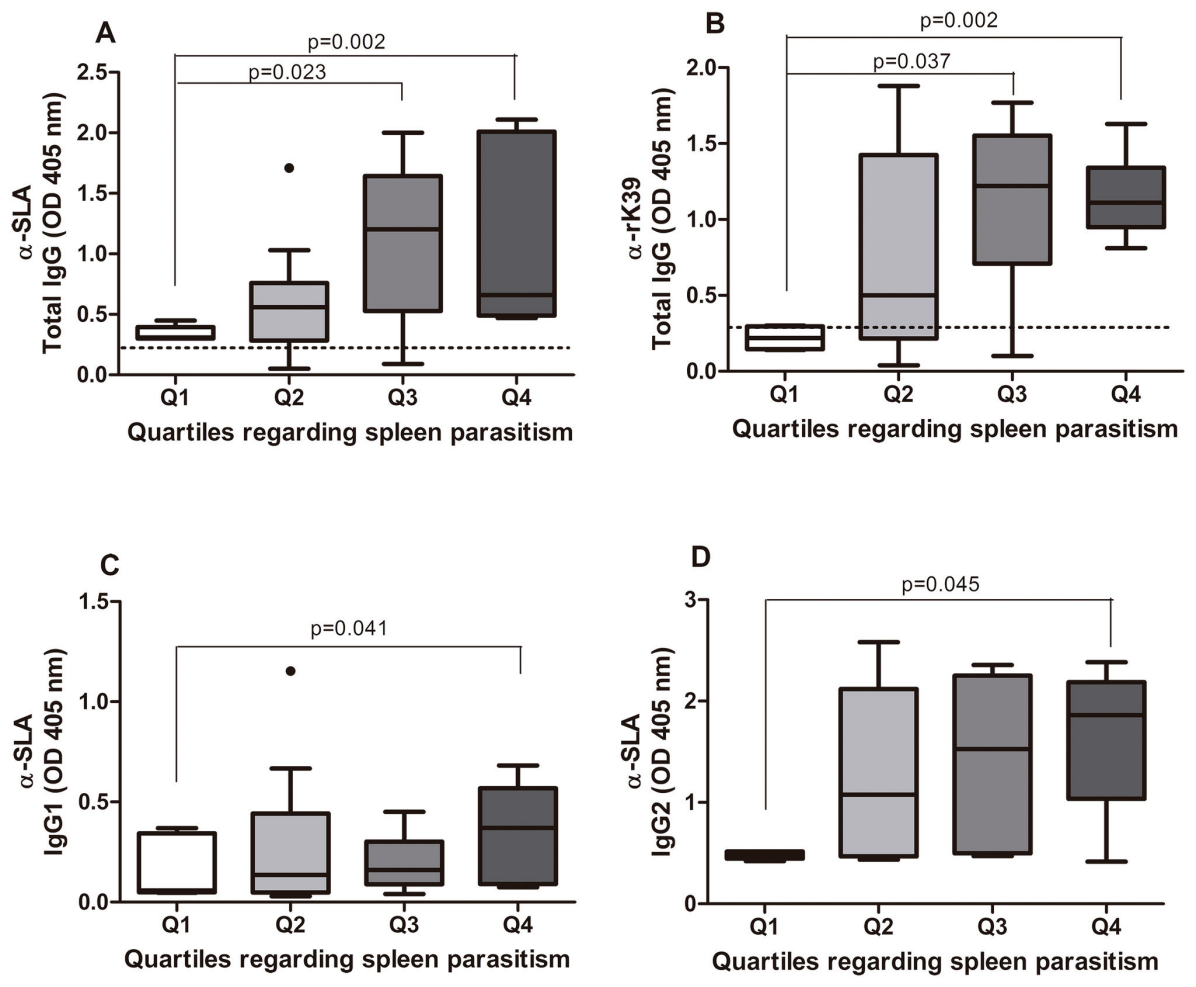
Anti-Leishmania antibodies in accordance to *Leishmania* load in the spleen. A. Total anti-*Leishmania* IgG antibodies. B. Anti rK-39 antibodies. The cut-off values of 0.096 and 0.112 for SLA and rK-39, respectively, which corresponded to the mean of the results of five healthy dogs plus three standard deviations. The dashed line represents the cut off value for the test. C and D levels of IgG1 and IgG2 subclasses. Analysis was performed using One-way ANOVA or Kruskal-Wallis test. The Kolmogorov-Smirnov test was used to test the normality of the data.

**Figure 3:**
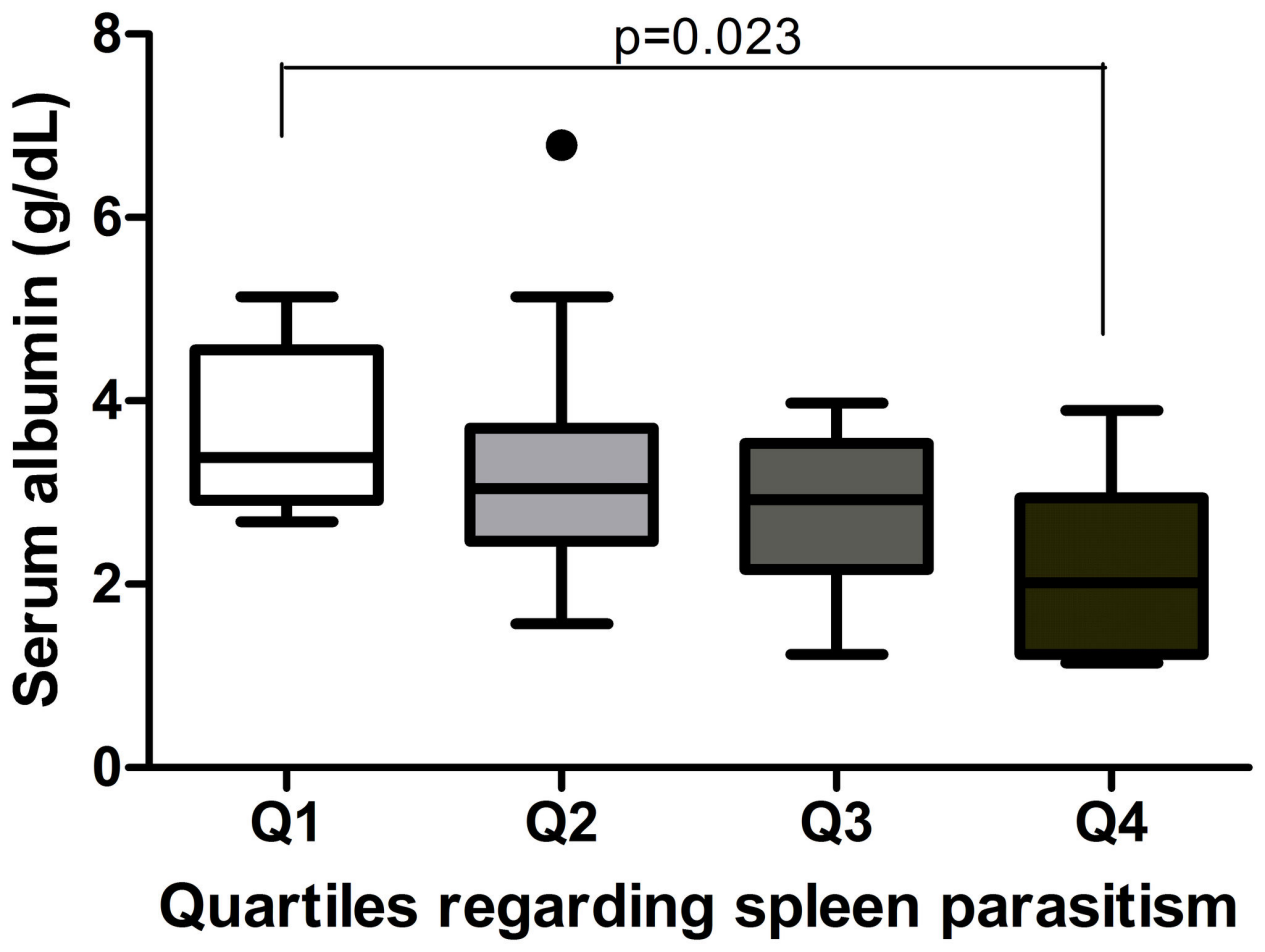
Sera albumin concentration in accordance to *Leishmania* load in the spleen. Analysis was performed by One-way ANOVA and the Kolmogorov-Smirnov test was used to test the normality of the data.

Dogs were grouped in quartiles in accordance to the quantity of *Leishmania* in the spleen, as presented on Figure 1B. Animals from quartile 1 were apparently healthy with mild alopecia; whereas dogs in quartile 2 the main had weight loss and moderate skin wounds. Dogs grouped in quartile 3 showed severe weight loss and conjunctivitis and, lastly, those in quartile 4 presented severe weight loss, pronounced onychogryphosis, alopecia, conjunctivitis, disseminated skin wounds and ascites. Of interest, levels of parasites in the blood were higher in female dogs, but there was no difference in the spleen burden between female or male. Sera albumin was higher in males (Table 1). An increase in anti-*Leishmania* antibodies was observed with the increase in *Leishmania* in the spleen (p=0.002, Figure 2A). Similar findings were observed for rK39, (p=0.002; Figure 2B). IgG1 was higher in animals from quartile 4 (Figure 2C), by IgG2 augmented with the increase in the *Leishmania* load (p=0.045; Figure 2D). IgG2 was a marker of symptomatic VL. Dogs with lowest spleen parasitism showed higher sera albumin concentration (3.66±0.94 g/dL), whereas animals from quartile 4 had lower albumin (2.19±0.95 g/dL), (p=0.023; Figure 3). 

**Table 1:** *Leishmania* load, anti-*Leishmania* antibodies and albumin in accordance to the sex of the dogs.

Sex	*Leishmania* load estimated by qPCR		OD 405 nm		Albumin (g/dL)*
	Spleen mg (mean ± SD)	Blood mL (mean ± SD)	SLA Mean ± SD	rK-39 Mean ± SD	Mean ± SD
Male	182,024±144,147	117,770±25,791	0.81±0.62	0.92±0.52	3.28±1.14
Female	212,310±193,300	218,226±23,518	0.99±0.67	0.88±0.68	2.41±1.11
Total	200,315±193,300	169,286±65,598	0.9±0.64	0.9±0.6	2.91±1.14
p value	0.8320	012	0.491	0.545	0.008

* Reference values for serum albumin concentration in dogs ranges from 2.3 to 4.5 g/dL of blood

### Cytokine gene expression in the spleen

**Figure 4:**
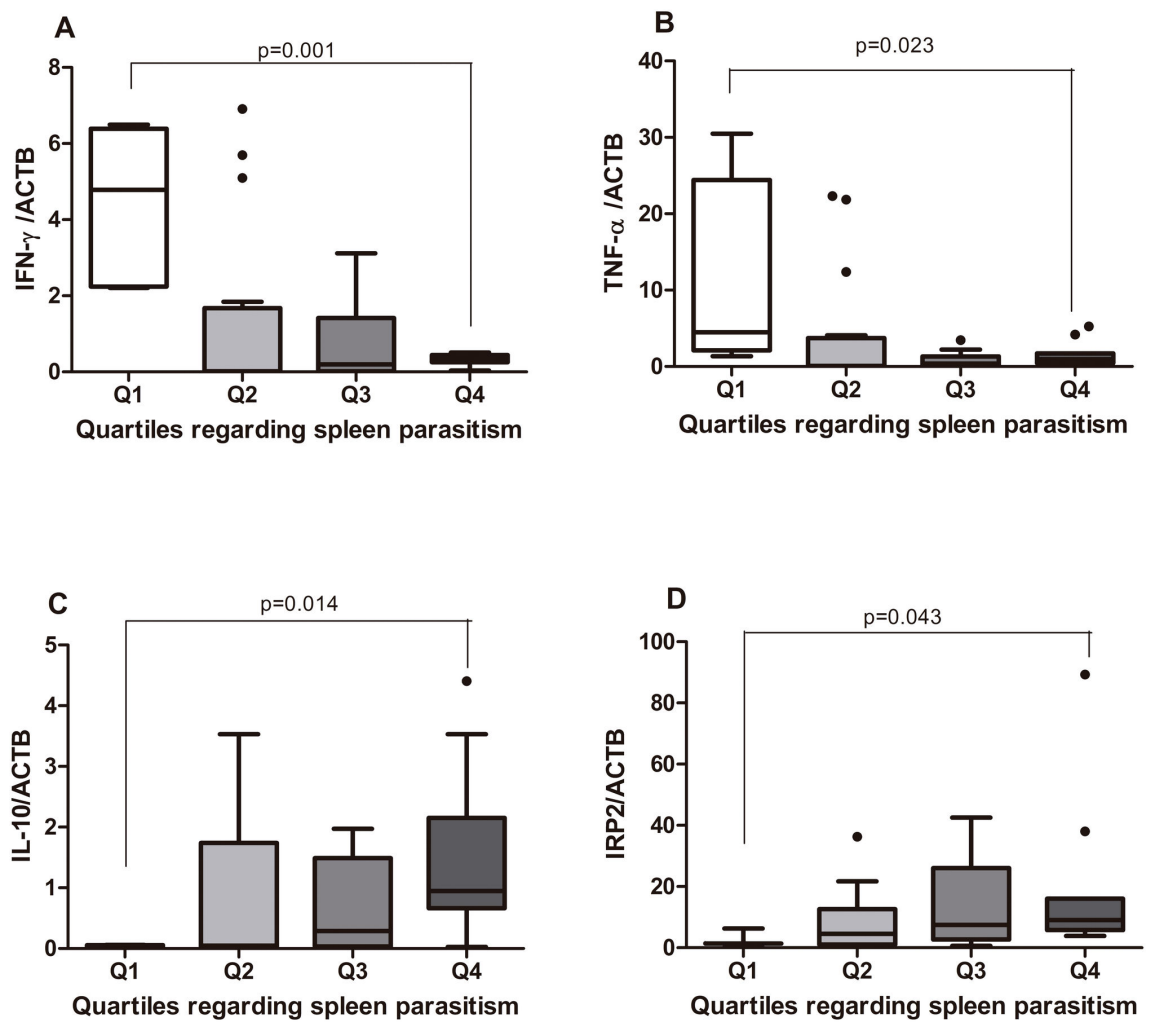
Spleen gene expression of cytokines and IRP2 in accordance to to *Leishmania* load. **A**, IFN-γ.B, TNF-α.C, IL-10 and **D**, IRP2. ACTB was taken as housekeeping gene and the first quartile was used as the calibrator group, since it presented the lowest spleen parasitism estimated by kDNA. Analysis was performed by One-way ANOVA or Kruskal-Wallis test and the Kolmogorov-Smirnov test was used to test the normality of the data.

Dogs belonging to quartile 1 expressed more *IFN-γ* and *TNF-α* in the spleen than animals in the last quartile (p<0.05, Figure 4A and B, respectively). A strong positive correlation was found between *IFN-γ* and *TNF-α* expressions in the spleen, (r=0.999, p=0.001), indicating a synergism between these cytokines function. Conversely, IL-10 expression increased with the increase in parasite load (p=0.014; Figure 4C). A negative correlation between *IL-10* and *IFN-γ* (r=-0.996, p=0.001) and *TNF-α* (r=-0.999, p=0.001) were observed. A moderate negative correlation was seen between *IFN-γ* and IgG2 (r=-0.475, p=0.002). Serum albumin (β=5.244; p=0.037) and *IFN-γ* (β=4.717; p=0.029) explained 22.8% of the variation in the spleen parasitism (Table 2). The model identified IFN-γ as the explanatory cytokine since TNF-α and IL-10 correlated, respectively, directly and inversely with IFN-γ expression. 

**Table 2:** Linear regression model considering the expression of the cytokines and parasite burden in the spleen of dogs naturally infected with *Leishmania*.

Explanatory variables	Ct k-DNA spleen			
	β	Std. error	t	p
(Constant)	14.477	1.976	7.327	0.000
Albumin concentration	5.244	2.418	2.168	0.037
IFN-γ gene expression	4.667	2.141	2.180	0.036

### Iron regulatory protein 2 (IRP2) expression

IRP2 is one of the proteins involved in the intracellular iron homeostasis. Intriguingly, dogs with elevated spleen *L. infantum* burdens expressed significantly more *IRP2* than animals with lower parasitism (p=0.043; Figure 4D). *IRP2* expression correlated directly to spleen parasitism (r=0.331, p=0.034) and *IL-10* expression (r=0.502, p=0.001) and inversely to *IFN-γ* (r=-0.474, p=0.002) and *TNF-α* (r=-0.487, p=0.001). 

### IFN-γ, TNF-α and IL-10 serum levels

**Figure 5:**
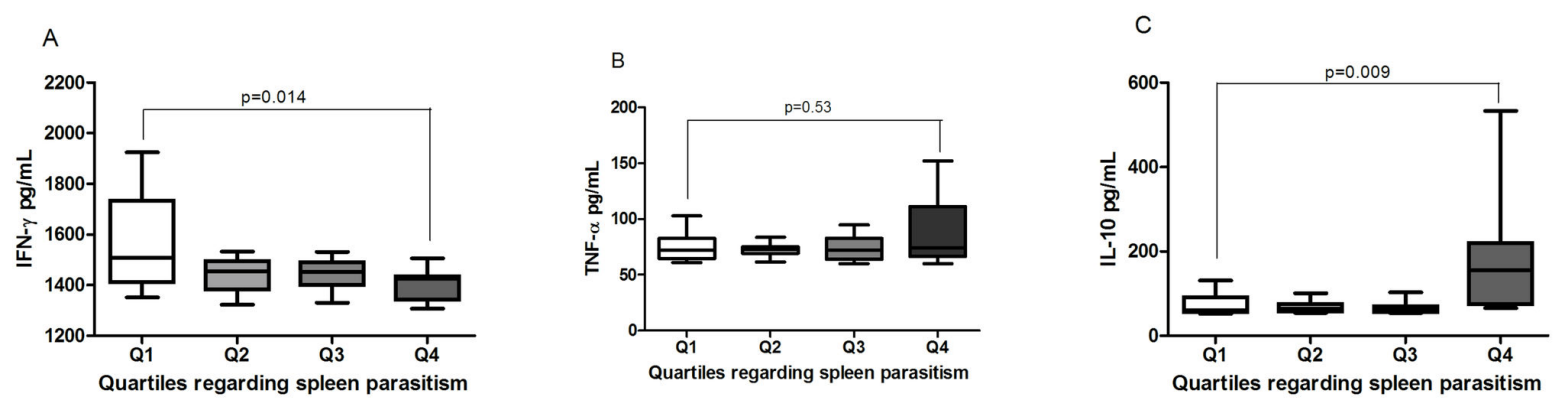
Levels of serum cytokines according to spleen *Leishmania* parasitism. **A**, IFN-γ, **B**, TNF-α and **C**, IL-10. Serum samples from each animal were used to quantify the concentration of these cytokines by ELISA and a standard curve was built using recombinant canine cytokine. Analysis was performed by One-way ANOVA or Kruskal-Wallis test and the Kolmogorov-Smirnov test was used to test the normality of the data.

Dogs with the lowest spleen parasitism (1st quartile) had higher IFN-γ serum level when compared to those with highest parasitism (p=0.014; Figure 5 A). This latter group (4th quartile) had the highest sera IL-10 level (p=0.009; Figure 5 C). TNF-α concentration was elevated, but there was no difference among the groups, (Figure 5B), tough TNF-α gene expression was elevated in the first quartile.

## Discussion

*L. infantum* infection causes a wide range of clinical outcomes in humans and dogs which are associated with several factors including the host immune responses, parasite polymorphisms, exposure to the sand fly vectors, and co-infections [25,26]. In this study, we found a negative correlation of *Leishmania* load with *IFN-γ* and *TNF-α* expressions and a positive correlation between *IL-10*. Leishmania load was lower in the blood than in the spleen, corroborating findings by Teixeira Neto et al [27], which showed increase parasitism with the severity of the *Leishmania* infection. Female dogs showed more parasites in the blood than male dogs, and this was associated with lower serum albumin. The increase in parasite circulation in female dogs could be related to an increased malnourished status due to multiple pregnancies and breast feeding. In this way, female dogs in the endemic areas for VL could be a more effective reservoir of *L. infantum* than males, but xenodiagnoses needs to be carried to assess this hypothesis [28]. These findings might have implications for the role of dog as reservoir, since levels of anti-*Leishmania* antibodies or circulating parasites can be low, but yet dogs can harbor high load of *Leishmania* in the spleen. In addition, since female dogs present high circulating *Leishmania* in the blood, these parasites could be easily transmitted via placenta to the fetus, as documented in dogs raised in kennel in the United States, without travel history to VL endemic areas [29]. In this way, vertical transmission could explain, in part, the high infection rate of *Leishmania* in some areas of Brazil, in spite the lower density of sand flies in some months of the year [30]. 

Pro-inflammatory cytokines as IFN-γ and TNF-α were highly expressed in the spleen of dogs with low parasitism, whereas, IL-10 was more expressed in animals with higher *Leishmania* burden, suggesting that the balance between these cytokines contribute to control of parasite multiplication and clinical outcome. Similar findings were seen for human VL in India [31]. In this way, these data strengthen the hypothesis that spleen parasitism is regulated by immune response in this organ and the ability of the animal to mount an effective Th1 profile response can determine whether *L. infantum* infection will evolve asymptomatically or to overt disease. Study showed that cytokine expression and immunoglobulin subtypes varied according to the range of infection of *L. infantum* infection [32]. Animals with sub-clinical infection expressed more IFN-γ and TNF-α than those with visceral leishmaniasis; in the same study, it was shown that animals with clinical manifestations had more parasites in the lymph node than those with asymptomatic infection. Differences in cytokine expression among organs have been shown in experimental leishmaniasis [33]. The difference between IFN-γ and L-10 explained the differences in parasite load between organs. A recent study carried out by Coura-Vital and co-workers [34], showed that asymptomatic dogs had a higher frequency of CD4 and CD8 T cells, these cells are the main producer of IFN-γ and TNF-α, which may lead to parasite control and maintenance of asymptomatic status. 

Iron plays an important role in cell homeostasis, it is a component of haem groups and integrate mitochondrial iron-sulphur cluster in proteins involved in electron transport and ATP production [35]. The role of iron on immune response in leishmaniasis and other infection is still not entirely understood. We then hypothesized that pro-inflammatory cytokines could reduce cellular iron intake by down modulating transferrin receptor expression, although a set of mechanisms act simultaneously to regulate intracellular iron content, as hepcidin and ferroportin pathway. In our study, we observed that *IRP2* expression was up regulated in animals with high *Leishmania* burden. Recent study showed that the knockout of LIT1, an iron transporter in *L. amazonensis*, led to inhibition of *Leishmania* replication [36]. Our study showed that dogs in the first quartile had less *IRP2* expression and higher *IFN-γ* and *TNF-α* expressions, whereas animals in the quartile 4 had increased expression of *IRP2* and increased expression of *IL-10* expression. Olakamni et al. [37] showed that pro-inflammatory cytokines, as IFN-γ, reduced transferrin receptor expression in *Mycobacterium tuberculosis*-infected macrophages, with reduction in cellular iron intake. These findings suggest that pro-inflammatory cytokines could effectively control parasite replication by an iron dependent manner.

Dogs with higher spleen parasitism had significantly less albumin in the sera. In addition, the analysis of *IFN-γ* expression explained differences in spleen parasite burden. The signaling pathway for IFN-γ is well characterized [38] and the blocking of parasite replication can be reached by host macrophage activation through this cytokine. In this way, the ability to effectively maintain a pro-inflammatory response, as IFN-γ and TNF-α, in the spleen could decrease parasite replication and, as a consequence control disease progression. Finally, IRP2 could influence the availability of iron to the parasite by an increment in the intracellular iron content, which seems to be inhibited by IFN-γ and augmented by IL-10.

## Supporting Information

Table S1 **Part number of each TaqMan® Assay used to determine cytokine expression.**
(DOCX)

Table S2 **Estimation of spleen parasite burden according to Ct found for each quartiles.**
(DOCX)
