# Supplementary material for: Association of Pro-Inflammatory Cytokines and Iron Regulatory Protein 2 (IRP2) with Leishmania Burden in Canine Visceral Leishmaniasis
Source: PLoS One. 2013 Oct 11;8(10):e73873. doi: 10.1371/journal.pone.0073873 (PMC3795717; doi:10.1371/journal.pone.0073873)
Supplement: Table S1 — Part number of each TaqMan® Assay used to determine cytokine expression. (DOCX) [file pone.0073873.s001.docx]

Table S1. Part number of each TaqMan® Assay used to determine cytokine expression.

| Gene ID | Assay ID |
| --- | --- |
| IFN-γ | Cf02623316_m1 |
| TNF-α | Cf02628237_m1 |
| IL-10 | Cf02624265_m1 |
| IRP2 | Cf02640728_m1 |
| ACTB | Cf03023880_g1 |
