# Supplementary material for: Association of Pro-Inflammatory Cytokines and Iron Regulatory Protein 2 (IRP2) with Leishmania Burden in Canine Visceral Leishmaniasis
Source: PLoS One. 2013 Oct 11;8(10):e73873. doi: 10.1371/journal.pone.0073873 (PMC3795717; doi:10.1371/journal.pone.0073873)
Supplement: Table S2 — Estimation of spleen parasite burden according to Ct found for each quartiles. (DOCX) [file pone.0073873.s002.docx]

Table S2. Estimation of spleen parasite burden according to Ct found for each quartiles.

| Quartile | Ct mean±SD | Parasites / spleen mg  mean±SD |
| --- | --- | --- |
| Q1 | 26.84±1.2 | 27,024±7,826 |
| Q2 | 23.81±2 | 84,399±36,524 |
| Q3 | 18.47±1.65 | 205,074±54,172 |
| Q4 | 14.07±1.22 | 485,192±125,661 |
